# Headache in Pregnancy: A Nuisance or a New Sense?

**DOI:** 10.1155/2012/697697

**Published:** 2012-02-15

**Authors:** Archana Dixit, Manish Bhardwaj, Bhavna Sharma

**Affiliations:** ^1^West Middlesex University Hospital, Twickenham Road, Isleworth, Middlesex TW7 6AF, UK; ^2^ST6 Anaesthesia, John Radcliffe Hospital, Oxford QX3 9DU, UK; ^3^Lewisham Hospital, London SE13 6LH, UK

## Abstract

Headache is a very commonly encountered symptom in pregnancy and is usually due to primary headache disorders which are benign in nature. It can however be quite debilitating for some women who may need therapeutic treatment of which there are several options safe to use in pregnancy. It is equally important though to recognise that headache may be a sign of serious underlying pathology. This paper aims to provide a clinically useful guidance for differentiation between primary and secondary headaches in pregnancy. The primary headache disorders and their management in pregnancy are explored in depth with brief overviews of the causes for secondary headaches and their further investigation and management.

## 1. Introduction

Headache is a very common condition and the commonest reason for referral to a neurologist [1]. Most headaches have a female predominance with peak incidence in the second and third decades, peak prevalence being in the fourth decade [2–6]. More than 80% of women in the reproductive age group experience headache at some stage making it a common symptom encountered during pregnancy [7].

Most headaches in pregnancy are benign and may cause a trivial inconvenience to quite significant debilitation. However, headache in pregnancy may herald the onset of life-threatening conditions such as eclampsia, stroke (haemorrhagic or thrombotic), and Arterio-Venous malformations (AVMs).

So is headache in pregnancy just a “*nuisance*” or could it be a “*new sense*” heralding the onset of a potentially life-threatening pathological process? The challenge is to identify the women who need further investigations in order to avoid significant morbidity and/or mortality.

**Classification**

Headaches in pregnancy may be classified as follows.

Benign, for example, migraine, tension-type headache, cluster headaches, analgesic-overuse headaches, and so forth.
Pathological, from an underlying pathology, for example, a vascular event (haemorrhage or thrombosis) or raised intracranial pressure (ICP) such as in brain tumours and benign intracranial hypertension.

The International Headache Society (IHS) has published a comprehensive classification system for headaches—The International Classification of Headache Disorder (ICHD) (see Table 1) [8].

## 2. Primary or Benign Headaches in Pregnancy

### 2.1. Migraine

A primary headache disorder defined by the IHS as headache with particular features—usually unilateral, often throbbing, and associated with nausea and sensitivity to light, sound, and head movement [8]. It is classified in 2 ways: migraine without aura (70%) and migraine with aura (30%) with the absence or presence of associated visual or neurologic phenomena called “aura” discriminating them. Aura can precede the headache, occur during it or afterwards. It is thought to result from a reduction in cerebral blood flow due to neuronal dysfunction rather than ischaemia.

Migraine is common, prevalence 12–15% in the western world [9]. It is three times more common in females than males in the postpubertal group [2, 9] while, prepuberty the sex ratio is 1 : 1 [10]. In females, the highest prevalence is during the childbearing years with peak (27%) at age 41. About 70% of migraineurs have a positive family history so genetic factors probably play a role in its onset.

#### 2.1.1. Pathophysiology of Migraine

The pathophysiology of migraine headaches is poorly understood with no one theory fully accounting for their varied presentations and symptomatology. The headache of migraine occurs from vasodilatation or oedema of mainly extracranial and meningeal arteries, with stimulation of perivascular nerve endings that stimulates the trigeminal system which in turn causes headache.

Release of vasoactive substances and complex interplay between several other neurotransmitters is thought to play a role: the inhibitory neurotransmitters, which decrease at headache onset like endorphins, serotonin (5-HT), and gamma amino butyric acid (GABA), and the excitatory neurotransmitters, which increase during headache episode like norepinephrine and dopamine. These changes in neurotransmitters cause dilatation of meningeal blood vessels.

 Serotonin depletion can induce migraine and selective serotonin agonists such as the triptans can alleviate it [11]. Migraine pathology occurs in brain structures such as the trigeminal pain pathway, brainstem nuclei, cerebral vasculature, thalamus and the primary sensory cortex [11–13].

*Migraine with Aura* occurs from a combination of neuronal disturbance and vascular dysfunction. Initially, abnormal neuronal activity in the hypothalamic and limbic systems leads to the prodromal symptoms lasting a few hours to a few days [14]. These may be neurological, psychological, or constitutional with 60% of migraineurs recognizing these as markers of an oncoming attack [15]. This prodrome is followed by aura—a wave of neuronal activation then inhibition emanating from a focus likely in the occipital cortex [16] accompanied by a decrease in cerebral blood flow leading to clinically apparent visual, sensory or motor phenomena with occasional brain stem and language disturbances. As aura subsides, a marked increase in blood flow occurs, in part due to serotonin-induced midbrain stimulation [17]. This is accompanied by a headache which is generally unilateral, throbbing, and associated with nausea and vomiting in up to 30% of patients [18] although it may be bilateral. Also seen is sensory excitability with symptom exacerbation by physical activity, light, sound or smell. The postdrome follows which less well characterised, cerebral blood flow is returning to normal during this time. Clinically the migraineur feels tired, lethargic, and depressed but occasionally has euphoria.

*Migraine without aura* seems to lack the phase of reduction in cerebral blood flow hence the absence of aura.

#### 2.1.2. Effect of Pregnancy on Migraine

Fluctuations in oestrogen levels are known to influence migraines with high oestrogen levels improving and falling levels deteriorating the symptoms [7] this being particularly apparent during the menstrual cycle with headache most likely to occur 2 days before or after the onset of menses [19]. A number of studies have shown that the high stable oestrogen levels in pregnancy improve migraine symptoms with up to 11% of women reporting improvement in the first trimester, rising to 53% in the second and 79% in the third trimester particularly women with menstrual migraine [7, 20, 21]. Conversely, others have found that the greatest improvement in headaches is seen in the first trimester and migraines that persist into the second trimester are less likely to improve [7]. Postpartum, the oestrogen levels rapidly fall explaining the exacerbation seen postpartum with up to 34% of women suffering a relapse in the first week rising to over 55% within a month [21]. Breastfeeding provides some protection probably relating to stable though low oestrogen levels.

Hormonal influences may not be the only reason for symptom improvement in pregnancy and analgesic effects of increased *β*-endorphins, and emotional changes may account for some cases of improvement.

4–8% of women experience deterioration in their symptoms migraine with aura less likely to improve for reasons that remain largely unexplained [22] though platelet hyperaggregation is suggested. This may also account for the increased risk of ischaemic stroke associated with migraine with aura. Some women develop migraine for the first time in pregnancy leading to a diagnostic dilemma.

#### 2.1.3. Effect of Migraine on Pregnancy

Migraines with or without aura, in general, do not seem to have any adverse effects on the outcome of pregnancy [23]; however, an increasing body of evidence suggests an association between migraine, preeclampsia, and eclampsia [24–27]. A recent case control study [28] found that a history of migraine was associated with a 1.8-fold increased risk of preeclampsia (95% CI 1.1–2.7). Older women (≥30 years) when diagnosed with migraine were at the highest risk (OR 2.8, 95% CI 0.8–9.0). Overweight migraineurs had a 12-fold increased risk of preeclampsia as compared to lean migraineurs (95% CI 5.9–25.7).

There is an increased risk of stroke in pregnant women [29, 30] and migraine particularly migraine with aura is an independent risk factor for stroke, particularly among young women [31]. However, not many studies have focused on the risk of pregnancy-related stroke in migraineurs. James et al. [32] reviewed data from the Nationwide Inpatient Sample relative to pregnancy-related discharges and found an overall risk of pregnancy-related stroke of 34.2 per 100,000 deliveries. The strongest associations for stroke were migraine with an OR of 16.9 (95% CI 9.7–29.5) and thrombophilia with an OR of 16.0 (95% CI 9.4–27.2).

#### 2.1.4. Management of Migraine in Pregnancy

The major concern in the management of the pregnant patient with migraines, as with other medical disorders, is the effect of both the medication and the disease on the fetus. As most women with migraines improve after the first trimester, they can usually be managed with reassurance and nonpharmacological measures such as ice packs, massage, relaxation, and biofeedback [33, 34]. A holistic approach yields the best results with avoidance of triggers, lack of sleep, and psychological stress. A lifestyle of regular exercise, regular meal times, and regular sleep patterns helps; later by reducing 5-HT-mediated neuronal activation [35].

Some women, however, continue to have debilitating and intractable headaches associated with nausea and vomiting with the attendant risk of dehydration. These women require pharmacological treatment.

The pharmacological treatment of migraines can be divided into abortive treatment—to manage an acute attack and prophylactic treatment—to prevent recurrence. Adjuvant therapy with antiemetics is commonly needed during acute attacks (see Table 2).

### 2.2. Abortive Therapy

*Paracetamol* is the first-line treatment for short-term relief of mild-to-moderate pain in pregnancy due to its safety in pregnancy. Its mechanism of action is poorly understood; however, it is an effective analgesic reducing the intensity though not the duration of pain in an acute attack of migraine [36].

*Opioids* modulate pain by suppressing activity in ascending nociceptive pathways and altering central perception by reducing primary nociceptive afferent activity [37]. Codeine phosphate is most commonly used and works better in combination with paracetamol [38]; however, daily opioid use should be avoided to prevent the development of medication-overuse headache. There is no increase in the incidence of birth defects in women given codeine during pregnancy despite earlier studies suggesting this [39] although transient symptoms of neonatal withdrawal may be seen after regular exposure in the third trimester.

*Antiemetics* are extremely useful adjuncts for the associated symptoms such as nausea and vomiting that can be equally disabling as the headache. Also, some medications used to treat migraines can cause nausea. Metoclopramide is particularly useful as it reduces the gastric atony seen with migraine, increases small intestine peristalsis and enhances the absorption of coadministered medications [40]. Its antidopaminiergic effect may also help prevent migraine headache if used in the prodromal phase [15] or to abolish established headache if used later. Other antiemetics such as chlorpromazine, prochlorperazine, and promethazine, can all be safely used in pregnancy [41].

*Aspirin* and other *NSAIDs* control migraine pain by inhibiting cyclooxygenase activity and thus reducing prostaglandin production [42]. Analgesic doses of aspirin and other NSAIDs have been shown to be safe in the first and second trimesters of pregnancy [43]; however, chronic exposure or exposure to high doses after 30 weeks of pregnancy is associated with premature closure of ductus arteriosus and restriction of renal blood flow in some foetuses [44, 45]. Their use has also been linked to increased risk of necrotising enterocolitis and intraventricular haemorrhage in potentially viable preterm babies [46]. Use of high doses of NSAIDs in the third trimester has been linked with antepartum and postpartum haemorrhage [47], and hence the use of aspirin and other NSAIDs should be avoided in the third trimester and when a very preterm birth is anticipated.

To obviate the potentially harmful effects of high doses of NSAIDs, they can be given at lower doses in combination with paracetamol to achieve a synergistic effect, frequently in combination with caffeine, which is thought to potentiate their efficacy and increase the speed of onset of pain relief. *Caffeine* is also an analgesic through its action as a cerebral vasoconstrictor and may also increase pain tolerance through a poorly understood mechanism [48, 49]. Daily intake of up to 300 mg caffeine appears to be safe in pregnancy; intake beyond this may be associated with miscarriage and fetal growth restriction [50].

*Ergot derivatives *are highly effective for treatment of acute migraine attacks by blocking 5-HT receptors and causing widespread vasoconstriction. However, they cause uterine hypertonicity and vascular disruption increasing the risk of miscarriage [51]. There have been reports of a small number of babies born with injuries consistent with tissue ischaemia after ergot use in pregnancy [52]. The use of ergot and its derivatives is contraindicated in pregnancy.

*Triptans *such as sumatriptan, rizatriptan, and naratriptan abort migraine attacks by antagonising 5-HT receptors in the midbrain. The safety of triptans during pregnancy has yet to be established although data from a number of cohort studies, drug company surveillance, and prospective pregnancy registries are reassuring and confirm that inadvertent exposure to sumatriptan during pregnancy is not associated with adverse fetal effects [53–56]. However, the work to date is not sufficiently powered to detect a small increase in fetal anomalies, so although women inadvertently exposed to triptans should be reassured, triptans should be avoided during pregnancy. Two studies have suggested a link between exposure to sumatriptan in second and third trimesters and fetal growth restriction and preterm birth although the results were statistically insignificant [54, 55]. There is limited data available for other triptans and the continued use of these agents is not recommended during pregnancy unless no other treatment is effective.

*Magnesium sulphate* intravenously has recently been shown to be an efficient, safe and welltolerated agent in the treatment of migraine, more effective than placebo in reducing or abolishing pain and in reducing the associated symptoms of nausea and vomiting [57, 58]. As its maternal and fetal safety profile is well established from its use in preeclampsia-eclampsia, magnesium sulphate may prove to be a useful agent for management of acute migraine attacks in pregnancy. Further studies are needed, however, before its routine clinical use can be advocated.

Dexamethasone has been used sporadically in severe migraine unresponsive to other therapies [59]; however its routine use in pregnancy is not justified as there is little reliable published evidence on its efficacy and possible adverse maternal and fetal side effects [60].

### 2.3. Prophylactic Therapy

As recurrent attacks of migraine can be extremely debilitating, prophylactic therapy should be considered if there are more than 3 acute attacks per month.

*Low-dose aspirin *is suggested as first-line agent for migraine prophylaxis in pregnancy given its safety established by extensive study during pregnancy for prophylaxis of preeclampsia. One small trial of 28 pregnant women with frequent or severe attacks of migraine taking low-dose aspirin (75 mg) for migraine prophylaxis showed subjective improvement in 22 women [61]. The ability of aspirin to counteract the platelet activation of pregnancy is thought to be the rationale behind its use for this indication.

*Beta blockers *prevent cerebral arterial vasodilatation thereby reducing the frequency and severity but not the duration of migraine attacks [62, 63]. Beta blockers have been suggested to be associated with fetal growth restriction [64] which is thought to be a class effect and is possibly dose dependent; hence, the lowest effective dose of propranolol or metoprolol is suggested. As an alternative, labetolol (combined alpha and beta blocker) has been used with some success in pregnancy [65].

*Antidepressants *such as amitriptyline or mirtazapine reduce the severity, frequency and duration of migraine attacks by a mechanism involving central inhibition of 5-HT and histamine receptors [62, 66]. Amitriptyline is particularly effective in women for reasons which are not clear [62]. Low doses of amitriptyline (10–50 mg at night) appear to be adequate and safe for migraine prophylaxis. At higher doses used for depression, tachycardia, irritability, muscle spasms, and convulsions have been reported in neonates.

*Antiepileptics *such as sodium valproate are being increasingly used for migraine prophylaxis outside of pregnancy; however a significant increase in fetal malformations precludes its use in pregnancy.

*Other drugs*—Although data on pitozifen are limited, there are no reports of adverse outcomes during pregnancy. Others such as verapamil, although safe, have very limited efficacy while methysergide is contraindicated in pregnancy.

## 3. Tension-Type Headaches (TTHs)

TTHs are the most common headache types, lifetime prevalence 78% with a female: male ratio of 3 : 1. They are characterized by a generalized pressure or tightness in the head which is unaffected by activity. Diagnosis of TTHs is based on history and clinical examination after excluding alternative explanation for the symptoms. Their differentiation from migraine is usually straightforward due to the episodic nature and accompanying symptoms of migraine. It may, however, be difficult to distinguish these from secondary headaches.

### 3.1. Pathophysiology of TTH

This is even less clear than the pathophysiology of migraines. The mechanism seems to be similar to migraine with the involvement of the same neuroanatomic structures such as the trigeminal nucleus. Serotonin and endorphins are again thought to play a major role [67]. TTHs would be expected to improve during pregnancy as these neurotransmitters are influenced by female hormones, and this is supported by clinical studies although improvement is less marked than that seen with migraines [3, 7, 68].

### 3.2. Effect of Pregnancy on TTHs

Studies evaluating the course of TTHs in pregnancy are few and far between and show conflicting results. In one study, 67% of TTH patients showed no change in symptoms, 28% reported improvement, and 5% worsening [3], while in another study 50% women with TTH reported improvement with only 33% of migraineurs reporting the same [23]. Sample sizes of these studies are too small to draw reliable conclusions or to identify prognostic factors which might help in predicting which patients would improve in pregnancy and which not.

### 3.3. Effect of TTH on Pregnancy

There does not appear to be any association between TTH and adverse pregnancy outcomes.

### 3.4. Management of TTHs in Pregnancy

As psychological factors and musculoskeletal stresses in pregnancy have been implicated in the worsening of TTH in pregnancy, nonpharmacological therapies such as relaxation, stress management, and biofeedback are highly efficacious for the management of TTHs in pregnancy with no side effect to mother or fetus [69, 70]. Pharmacological treatment is similar to that of migraines with paracetamol and NSAIDs being the mainstay of acute treatment with the same guiding principles as discussed in the treatment of migraines. Prophylactic treatment is seldom indicated and only if headaches are occurring more than 2 to 3 days per week.

## 4. Cluster Headaches (CHs)

It is severe primary headache disorder accompanied with autonomic symptoms [8] such as ipsilateral nasal congestion and rhinorrhoea, lacrimation, conjunctival hyperaemia, facial diaphoresis, palpebral oedema, and Horner's syndrome. CHs are characterised by attacks of excruciating, penetrating pain typically short in duration (5–180 minutes) with no aura. The pain is classically periorbital but may radiate to other areas of the face and neck. Headache frequency ranges from once every other day to as often as several times a day [71]. Triggers include alcohol, vasodilator, drugs and sleep apnoea-induced hypoxaemia.

CH is relatively rare (prevalence 0.06 to 0.4%) even more so during pregnancy as this is one of the few headache disorders with a male predominance, male: female ratio 9 : 1 [72, 73]. Female hormones seem to have little influence on CH [74, 75].

### 4.1. Pathophysiology of CHs

Little is known about the pathogenesis of CHs. The periodicity of CHs has been attributed to hypothalamic hormonal influences with the suprachiasmatic nuclei and the posterior hypothalamic grey matter being the most likely site of pathology [76, 77]. The typical pain of CHs is thought to originate at the pericarotid/cavernous sinus complex which receives sympathetic and parasympathetic input from the brain stem, explaining the occurrence of autonomic phenomena during an attack [78]. The influence of hypoxaemia and hypocapnia in CHs is still unclear.

### 4.2. Pregnancy and CH

Due to the relative rarity of CH in women and its episodic nature, there are few studies of the effect of pregnancy on CH, the results of which are equivocal. Overall, pregnancy does not seem to have a major impact on CH and vice versa [74, 75].

### 4.3. Management of CH in Pregnancy

In the nonpregnant patient, 100% oxygen by face mask, corticosteroids, triptans and ergot alkaloids are the mainstay of abortive treatment for CH. CH may be particularly difficult to treat during pregnancy. 100% oxygen is safe to use and effective in about 70% of attacks [79]. Corticosteroids are compatible with use in pregnancy and lactation, but triptans should be used sparingly while ergot derivatives are contraindicated during pregnancy. Prophylaxis is rarely indicated with verapamil and corticosteroids being the most commonly used prophylactic medications [71].

## 5. Medication-Overuse Headache (Analgesic Rebound Headache)

It is important to remember this entity which describes the headache-precipitating tendency following frequent or long-term use of analgesics. Medication overuse may be due to a physical or psychological dependency with the prime quest being pain relief and is present in about 40% of migraineurs. Provoking agents include ergotamine, triptans, simple analgesics, opioids and combination analgesics. Management includes patient education, explanation of the diagnosis, and gradual withdrawal of the causative agent which improves symptoms with majority of patients reverting to their primary headache disorder within two months.

## 6. Secondary or Pathological Headaches in Pregnancy

Pregnant women presenting with new onset headache in the pregnancy or those with atypical features such as focal neurological signs, papilloedema, or seizures should be urgently evaluated for underlying secondary causes and a neurology opinion sought. The causes for secondary headaches in pregnancy include preeclampsia-eclampsia, stroke (haemorrhagic or ischaemic), cerebral venous thrombosis, benign (idiopathic) intracranial hypertension (BIH), pituitary apoplexy meningitis/encephalitis, postpartum cerebral angiopathy, and postpartum dural puncture headache.

### 6.1. Preeclampsia-Eclampsia

Headache is a symptom of severe preeclampsia and occurs in about 75% of women with eclampsia where it always precedes the seizure [80]. Headache in preeclampsia-eclampsia can be bitemporal, frontal, occipital, or diffuse with most women describing the pain as pulsating although a feeling of pressure or sharp pain is described by some. A characteristic feature is its progressive nature and failure to respond to over-the-counter (OTC) remedies. It can be associated with visual changes such as blurred vision, scotomata, or bright flashing lights [81]. The pathophysiology is largely unknown, but two aetiologies are suggested: (1) marked vasospasm in the cerebral vasculature in response to elevated systemic blood pressure resulting in ischaemia and (2) vasoconstriction of cerebral vasculature followed by reflex vasodilatation leading to overdistension, extravasation of fluid and cerebral oedema. Normally cerebral autoregulation protects against sudden changes in blood pressure; however, when systolic blood pressure exceeds 150 mmHg, the autoregulation starts to fail and hypertensive encephalopathy may develop [82].

### 6.2. Stroke in Pregnancy

Stroke occurs in 3.5 to 8.6 women per 100,000 deliveries [82, 83]. One study has shown a rate of ischaemic stroke of 11 per 100,000 deliveries and intracerebral haemorrhage of 9 per 100,000 deliveries, greatest risk of both in the postpartum period [83]. Headache occurs with acute stroke in 18% to 38% of patients, more so in haemorrhagic than in ischaemic stroke [84, 85]. Epidemiological studies have identified a relative risk of stroke during pregnancy as 2.4 or an excess risk of 8.1 strokes per 100,000 pregnancies [29]. The mortality following pregnancy-related stroke is estimated at 10–13% being disproportionately higher in black women, older women, and those with no antenatal care [86, 87].

### 6.3. Ischaemic Stroke

Risk factors for ischaemic stroke in pregnancy include African-American ethnicity, age over 35 yrs, caesarean delivery, known medical disorders such as hypertension, diabetes, sickle cell disease, vasculitis, antiphospholipid syndrome or thrombophilia, heart disease and SLE, smoking, alcohol and recreational drug abuse (particularly cocaine), multiple gestation, and multiparity. Complications of pregnancy such as preeclampsia-eclampsia, hyperemesis, and disturbances in electrolyte and fluid balance are other significant risk factors. In one study, 47% of non-haemorrhagic pregnancy-related strokes occurred in the context of preeclampsia-eclampsia [83].

Diagnosis is by MRI or CT to confirm and also to differentiate haemorrhage from infarction. Treatment depends on the underlying cause, and antiplatelet therapy and/or anticoagulation may be needed. The safety of acute thrombolysis in pregnancy remains unproven, but there are case reports of its use with no adverse effects [88–90].

### 6.4. Haemorrhagic Stroke

This is very rare in women of child-bearing age outside pregnancy but almost as common as ischaemic stroke during pregnancy. Eclampsia accounts for 44% of intracerebral haemorrhagic strokes in pregnancy [83]. In one study, of 34 cases of pregnancy-related stroke, 13 were haemorrhagic with 7 being subarachnoid haemorrhage (SAH) [91]. SAH, resulting from aneurysms or arteriovenous malformations (AVM), accounts for 3% of all strokes [92]. Outside pregnancy, the ratio of aneurysm to AVM is 7 : 1, but, in pregnancy, relatively more cases are due to AVMs (ratio 1 : 1). A rare cause of intracerebral haemorrhage in pregnancy is choriocarcinoma, metastases of which are frequently haemorrhagic so may present in pregnancy with an intracerebral or SAH [93].

Risk of haemorrhage from an AVM in previously asymptomatic women is around 3.5% [94]. AVMs can dilate under effect of oestrogen. Pregnancy does not seem to increase the risk of first haemorrhage from an AVM; hence, it would seem reasonable to defer treatment of an AVM that has not bled until after delivery; however, if diagnosed before pregnancy, pregnancy should be deferred until after treatment. In patients who present with a bleed during pregnancy, decision for interventional treatment is more difficult with currently endovascular intervention preferred over surgery [92]. There is no advantage of a caesarean section over vaginal delivery; however, it is prudent to endure adequate pain relief with epidural analgesia and to curtail the length of the second stage of labour.

The overall risk of recurrence of stroke in a subsequent pregnancy is small.

### 6.5. Cerebral Venous Thrombosis (CVT)

uncommon (incidence 1 : 10,000) with most cases occurring postpartum with a high mortality rate [95]. Underlying pathology is thought to be the hypercoagulability of pregnancy; however, a thrombophilia screen is recommended to rule out any additional prothrombotic tendency. One report showed a link between puerperal CVT and Protein S deficiency [96]. Presenting symptoms include headache, seizures, altered consciousness, and neurological deficits with signs of raised ICP. The gold standard for diagnosis is venous angiography MRI. Management includes hydration, anticonvulsants, and anticoagulants. Although firm evidence for the benefit of anticoagulation is lacking, a number of studies during pregnancy have suggested improvement in survival following anticoagulation [97].

### 6.6. Benign (Idiopathic) Intracranial Hypertension (BIH)

Benign intracranial hypertension or pseudotumour cerebri is a unique syndrome seen mostly in obese young women, and pregnancy has been identified as a risk factor [98]. The volume of cerebrospinal fluid (CSF) is increased due to unknown reasons resulting in raised ICP leading to headaches and papilloedema. Symptoms of BIH usually increase during pregnancy [99].

Raised ICP may also result from *brain tumours*; however, these are rare during pregnancy and usually present with raised ICP, neurological deficits, or seizures rather than headaches. Pregnancy is associated with accelerated growth of some tumours such as meningiomas and pituitary adenomas [100].

### 6.7. Pituitary Apoplexy

Pituitary apoplexy during pregnancy is a rare but serious event with significant morbidity and possible mortality, if not timely recognised. It is a clinical syndrome consisting of sudden onset of severe headache, altered consciousness, vomiting, visual disturbances, and ophthalmoplegia thought to be caused by haemorrhage and/or infarction in the pituitary gland, although exact pathophysiology remains unknown. Its occurrence in pregnancy is rare with only a few cases reported. Usually a preexisting pituitary adenoma is present although it may be the first sign of a pituitary tumour. Oestrogens cause hyperaemia of the hypophysis and could therefore contribute to the risk of apoplexy in pregnancy [101]. Treatment consists of replacement of the deficient hormones especially glucocorticoids, close surveillance, and transsphenoidal surgery [102] but some advocate conservative management especially in pregnancy with surgery reserved for cases where there is no spontaneous improvement or worsening of visual impairment and/or consciousness [103].

### 6.8. Meningitis/Encephalitis

Any infection of the head/neck can present as headache but the diagnosis is likely to be suggested from the accompanying symptoms.

### 6.9. Postpartum Cerebral Angiopathy (PPCA)

It is cerebral dysregulation syndrome affecting large- and medium-sized cerebral arteries, it may be associated with haemorrhagic or ischaemic stroke usually within the first week postpartum. Although the pathophysiology is unclear, it is thought to be an inflammatory process such as vasculitis or transient vasospasm seen as areas of stenosis and ectasia in multiple intracranial vessels on cerebral angiography [104]. Clinical picture typically occurs in women aged 20–50 years with abrupt onset of severe “thunderclap” headaches, seizures, and focal neurological deficit [105]. Several medications, for example, ergotamine, triptans and bromocriptine, if given intra- or postpartum can contribute to its development by their vasospastic effect on cerebral vasculature [106]. Prompt diagnosis and treatment can prevent fatality. Treatment includes corticosteroids and calcium channel blockers such as nimodipine (preferential cerebral vasodilator). Most events resolve quickly without permanent neurological deficits.

### 6.10. Postpartum Dural Puncture Headache (PDPH)

PDPH is a disabling and distressing complication of inadvertent puncture of dural membrane while performing epidural analgesia in parturients. Excessive CSF seepage from the dural tear leads to a fall in CSF pressure and stretching of meninges manifesting as a headache. Compensatory intracranial vasodilatation in response to fall in CSF pressure may further worsen the headache. Incidence of unintentional dural puncture with epidural needle is around 1% while the incidence of PDPH following dural puncture with 16–18 G Tuohy needle is approximately 70%. Common features of PDPH are:

- onset 24–48 hrs post dural puncture,
- severe, throbbing headache in the frontooccipital region which can radiate to neck and shoulders,
- pain exacerbation by head movement and upright posture, relieved by lying down,
- associated features such as photophobia, nausea, vomiting, tinnitus, diplopia, hearing loss, dizziness and VIth cranial nerve palsy may be present.

Reassurance, hydration, simple analgesics, and caffeine are the mainstay of early treatment, while epidural blood patch (EBP) is the definitive treatment [107]. Mechanism of action of EBP is twofold (1) blood in epidural space compresses the dural sac raising ICP which provides immediate pain relief and (2) sealing of dural tear by blood clot preventing further CSF leak. EBP performed after 24 hours in symptomatic patients has a success rate of 80–90% increasing to 95% after second EBP. EBP should be avoided in the first 24 hours because of a high failure rate around 70% [108].

### 6.11. A Practical Approach to Management of a Pregnant Woman Presenting with Headache

Pregnant women with a headache can be categorized into three groups:

1. women with a known primary headache disorder presenting with their typical headache,
2. women with a known primary headache disorder presenting with a headache different in character from their typical headache,
3. women presenting with a new onset headache.

Even though migraine is known to change character in pregnancy, the first scenario is likely to be benign; however, in the other two, a high index of suspicion should be maintained for secondary causes and neurologists involved in the care. We suggest the following approach to management. 

Thorough history—A careful history can be diagnostic in most cases. Particular note should be given to “red flags” or “warning signs”—“*NEW SENSE”*: Table 3.

Clinical examination:
  a thorough general examination with vital signs,
  a full neurological examination including ophthalmologic examination for papilloedema.
Investigations:
  urinalysis,
  full blood count,
  blood Biochemistry including liver function tests,
  coagulation profile.
Additional Investigations:
  radiological imaging:
  CT and/or MRI without contrast,
  lumbar puncture:
  if fever/neck rigidity/raised white cell count,
  thunderclap headache with negative CT to
  r/o subarachnoid bleed,
  r/o benign intracranial hypertension.

## 7. Conclusion

Most headaches in pregnancy are benign and due to primary headache disorders being largely treatable with reassurance, nonpharmacological remedies, and simple analgesics. A high index of suspicion for serious underlying pathology should be maintained if atypical features are identified on history and/or clinical examination especially if a woman presents with a headache for the first time in her pregnancy in order to institute timely management and prevent significant morbidity and mortality.

## Figures and Tables

**Table 1:** International Classification of Headache Disorder (ICHD).

Part I: the primary headaches	
(1) Migraine—with or without aura	
(2) Tension-type headaches	
(3) Cluster headache and other trigeminal autonomic cephalalgias	
(4) Other primary headaches	

Part II: the secondary headaches	

(1) Headache attributed to head and/or neck trauma such as domestic violence, MVA	
(2) Headache attributed to cranial or cervical vascular disorder—hypertension, subarachnoid haemorrhage	
(3) Headache attributed to nonvascular intracranial disorder—raised intracranial pressure, meningitis	
(4) Headache attributed to a substance or its withdrawal—illicit drug use such as cocaine, alcohol or medication overuse headache	
(5) Headache attributed to infections	
(6) Headache attributed to disorder of homeostasis—hypoglycaemia, hypoxia	
(7) Headache or facial pain attributed to disorder of cranium, neck, eyes, ears, nose, sinuses, teeth, mouth or other facial	
or cranial structures—trigeminal neuralgia, Bell's palsy	
(8) Headache attributed to psychiatric disorder	

Part III: Cranial neuralgias central and primary facial pain and other headaches	

(1) Cranial neuralgias and central causes of facial pain	
(2) Other headache, cranial neuralgia, central or primary facial pain	

**Table 2:** Drugs commonly used for treatment and/or prophylaxis of migraine and their FDA rating and fetomaternal effects.

Drug	FDA rating	Maternal effects	Fetal effects	Compatible with breastfeeding
Paracetamol	B	Medication overuse	None	Yes
Opioids	B/C	Overuse/dependence Constipation	??cleft palate/inguinal hernia	Yes
Aspirin	C	Increased risk bleeding	Narrowing of ductus arteriosus	Yes
NSAIDs	B/D	Inhibition of implantation	Premature closure of ductus arteriosus Restriction of renal blood flow NEC and IVH in preterm foetuses	Yes
Triptans	C	Limited evidence possible increased miscarriage	?Preterm birth and IUGR	??
Antiemetics	B/C	None	None	Yes
Caffeine	B	Overuse/withdrawal headache	NoneHigh doses—miscarriage, IUGR	Yes
Ergot alkaloids	X	Uterine hypercontractility Reduced placental perfusion	Miscarriage Fetal compromise Intestinal atresia	No
Beta blockers	B/C	None	?IUGR, fetal bradycardia	Yes
Antiepileptics	C/D	None	Malformations	Yes
Antidepressants	B/C	None	High doses—neonatal depression, irritability, spasms, or convulsions	Caution

**Table 3:** Warning signs and symptoms—“*New Sense.” *

(i) New onset headache or change in pattern of chronic headache	
(ii) Neurological signs, for example, seizures, focal deficits, gait disturbances, visual changes, slurred speech, and so forth	
(iii) Meningeal signs	
(iv) Fever	
(v) Vomiting	
(vi) Headache changing with posture	
(vii) Thunderclap onset	
(viii) Valsalva headache (headache triggered with Valsalva-type manoeuvres)	
(ix) Trauma	
